# Statistical parametric mapping analysis of upper limb joint coordination and variability differences during the downswing phase of the golf swing

**DOI:** 10.3389/fspor.2026.1820927

**Published:** 2026-05-29

**Authors:** Yajun Xiao, Tianhao Zhang, Ruifeng Huang, Yong Ma, Weitao Zheng

**Affiliations:** 1 Key Laboratory of Sports Engineering of General Administration of Sport of China, Wuhan Sports University, Wuhan, China; 2 Research Center of Sports Equipment Engineering Technology of Hubei Province, Wuhan Sports University, Wuhan, China; 3 Engineering Research Center of Sports Health Intelligent Equipment of Hubei Province, Wuhan Sports University, Wuhan, China; 4 School of Physical Education, Wuhan University of Technology, Wuhan, China; 5 School of Fashion and Textiles, The Hong Kong Polytechnic University, Hong Kong, Hong Kong SAR, China

**Keywords:** continuous relative phase, golf swing, handicap level, joint coordination, statistical parametric mapping

## Abstract

**Objective:**

The aim of this study was to use statistical parametric mapping (SPM) to investigate differences in upper-body joint coordination and variability between golfers with low and high handicaps during the downswing phase.

**Methods:**

Fourteen right-handed male golfers each from the low and high handicap groups were recruited. Kinematic data were collected by Vicon 3D motion capture system. Visual3D software calculated joint angles and angular velocities. Customized Matlab scripts computed joint coordination and variability curves. SPM-based independent samples t-tests analyzed differences in upper limb joint coordination and variability during the downswing phase between groups.

**Results:**

In joint coordination, the low and high handicap groups differed in two couplings. In the left elbow (sagittal plane)-left wrist (sagittal plane) coupling, the low handicap group exhibited left elbow dominance, whereas the high handicap group showed left wrist dominance (*p* < 0.001). In the left shoulder joint (frontal plane)-left wrist joint (frontal plane) coupling, the low handicap group exhibited left shoulder dominance, whereas the high handicap group showed left wrist dominance (*p* < 0.001). Both findings remained statistically significant after Bonferroni correction. A between-group difference in coordination variability was observed only at the uncorrected level and did not remain significant after Bonferroni correction.

**Conclusions:**

(1) Regarding joint coordination, the high handicap group exhibited earlier wrist engagement in the early downswing and a more pronounced tendency toward inverse motion between adjacent joints. (2) Regarding coordination variability, the high handicap group showed lower variability in elbow-wrist joint coupling during a brief interval at the end of the downswing. However, as this result was no longer significant after Bonferroni correction, it should be interpreted cautiously as a preliminary finding.

## Introduction

The downswing phase is a critical component of the golf swing and one of the key factors determining shot performance (1–3). At the same time, the downswing phase is directly related to club delivery, impact execution, and the rapid generation of clubhead speed. The quality of downswing motion depends in part on the coordinated movement of the shoulder, elbow, and wrist joints (4, 5), which play a direct role in distal control of the club. Current research on the golf swing predominantly employs discrete indicators (such as peak angles and torques) for biomechanical analysis (6–8), which struggles to reveal the dynamic coordination characteristics across multiple joints. Therefore, investigating the multi-joint dynamic coordination features of the upper-body swing motion during the downswing for golfers with low and high handicaps can provide valuable insights for refining swing technique.

Continuous relative phase (CRP) is used to quantify the motion relationship between adjacent joints, i.e., joint coordination. It describes the covariation between the angles and angular velocities of the shoulder, elbow, and wrist joints during the swing motion, thereby revealing the temporal characteristics of joint coordination (9). Building upon this, the variability of continuous relative phase (VCRP) reflects the stability of joint coordination patterns (10). Higher coordination variability correlates with increased risk of sports injuries (11, 12). CRP has been applied in sports such as swimming (13), karate (14), and volleyball (15) to differentiate skill levels and coordination patterns. Concurrently, VCRP, as a key indicator of joint coordination stability, has been utilized in research on sports injury mechanisms (16, 17), validating the effectiveness of both measures in quantifying joint coordination movements.

However, the application of CRP and VCRP in studying the joint coordination system of the upper-body swing in golf remains under-explored, limiting deeper understanding of swing technique variations. Therefore, this study employed continuous relative phase to describe joint covariation in angle and angular velocity in a time-resolved manner, capturing the timing and magnitude of coordination patterns and their variability between joints. Given the continuous-time nature of CRP and its variability, statistical parametric mapping (SPM) provides a robust one-dimensional analytical framework. This approach enables point-by-point comparisons of time-normalized waveforms, controls for Type I errors, and identifies specific phase differences that traditional discrete analyses may overlook. This statistical method has been applied and validated in continuous biomechanical analyses of activities such as jumping and landing (18–20).

Therefore, this study quantified the coordination and variability of upper limb joints between golfers with low and high handicaps during the downswing stage, and used statistical parametric mapping analysis to explore the differences in upper limb joint movement patterns in time series and stability.

## Material and methods

### Participants

Sample size estimation was performed using power1d (v0.1.7) in Python 3.9 (21). Given the limited availability of directly relevant golf CRP/VCRP datasets, we extracted data from previous one-dimensional biomechanical studies of upper-limb tasks (22, 23) as approximate references. Data from these studies were digitized using WebPlotDigitizer v4.8 (https://automeris.io/WebPlotDigitizer), and interpolated to 101 points using spline functions. Based on prior literature data, we constructed the expected effect under the alternative hypothesis (H_1_) as a Gaussian pulse signal positioned at 1% (q = 1) with a width of 25 data points (fwhm = 25). Meanwhile, we estimated the noise standard deviation (sigma) as 5.0 units based on literature data, and assumed noise smoothness (fwhm) of 20.The null hypothesis (H₀) was defined as no effect (24). We employed independent-samples t-tests with a significance level of α = 0.05 and target statistical power of 0.8 as thresholds. Monte Carlo simulations were conducted with 10,000 iterations across different sample sizes. The analysis results indicated that a minimum sample size of 11 participants per group was required to achieve 80% statistical power. Considering the potential for data loss, 14 right-handed male golfers with low handicaps (3.15 ± 1.33) were recruited as the low handicap group (age 21.4 ± 1.78 years; height 177.3 ± 5.96 cm; weight 75.8 ± 9.83 kg). 14 right-handed male golfers with high handicaps (15.0 ± 3.5) were recruited as the high handicap group (age 21.8 ± 0.63 years; height 174.5 ± 4.25 cm; weight 71.9 ± 10.38 kg). The low handicap group exhibited significantly higher clubhead speed than the high handicap group (*p* < 0.05), as shown in Table 1.

**Table 1:** Overview of clubhead speed and ball speed in the low and high handicap groups.

Variable	Low handicap group (*n* = 14)	High handicap group (*n* = 14)	t	df	*p*
Clubhead speed (mph)	109.13 ± 6.39	100.76 ± 4.68	3.954	23.83	0.001
Ball speed (mph)	160.20 ± 10.25	141.70 ± 9.07	5.057	25.62	<0.001

All subjects had no history of musculoskeletal disorders within the past three months, and golfers experiencing any pain in joints or associated muscles were excluded from the study. This experiment was approved by the Medical Ethics Committee of Wuhan Sport University (Approval No.: 2023042), and all subjects were thoroughly informed about the experimental protocol and signed informed consent forms.

## Research methods

### Test instruments

Kinematic data of the swing motion was captured using a 3D motion capture system (Vicon, UK, sampling frequency 250 Hz, 9 T40 model 4-megapixel cameras). Clubhead speed data was collected via radar monitoring equipment (Trackman, Denmark, model 3e), which also assisted in evaluating the effectiveness of executing technical movements.

**Figure 1:**
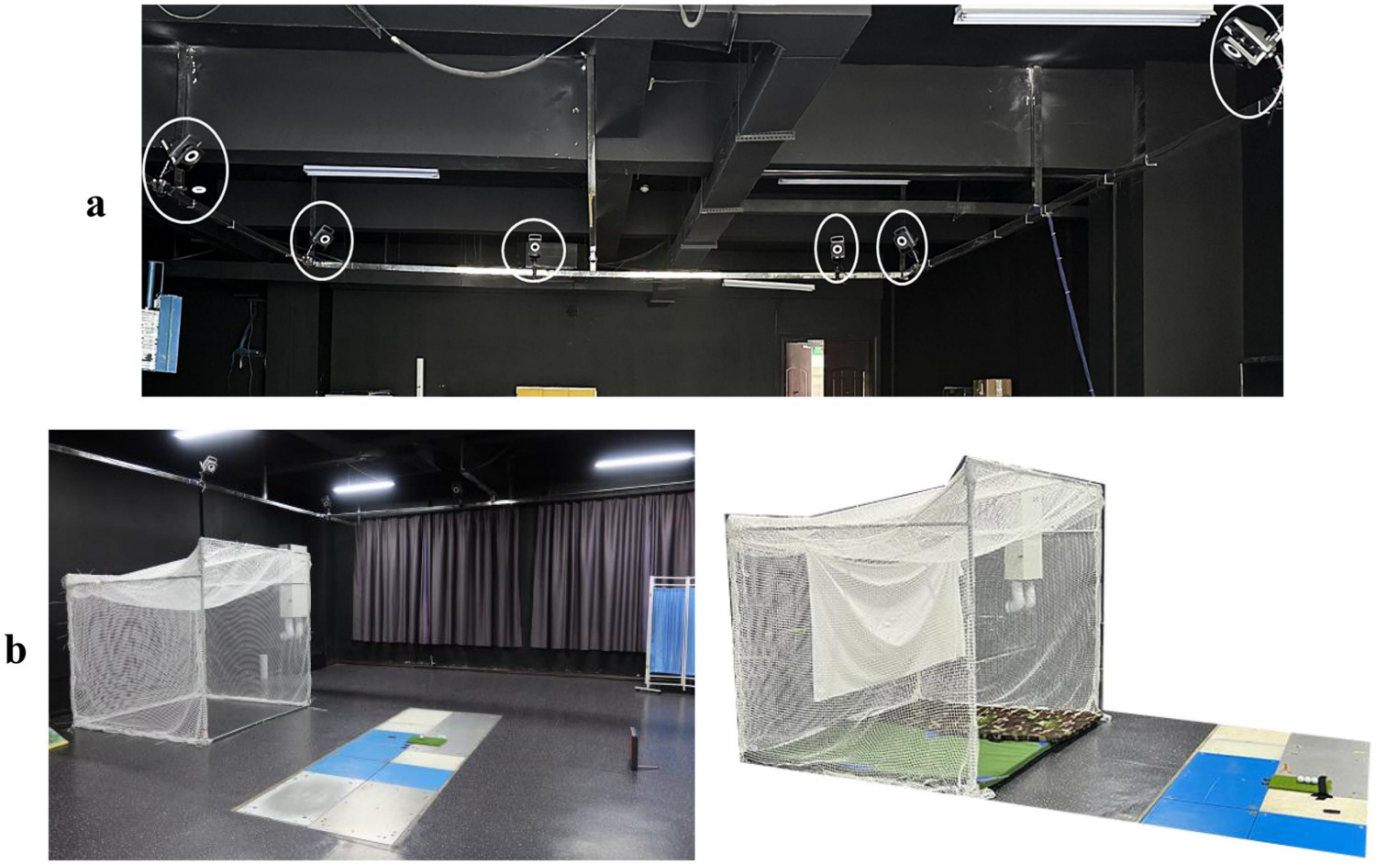
Testing equipment and environment **(a**: Vicon; **b**: testing environment).

A homemade galvanized steel pipe was used to construct a golf hitting cage (2 m long, 2 m wide, 2.1 m high), which was wrapped with architectural nylon safety netting with a mesh diameter of 2 cm. An impact-resistant fiber cloth was suspended at the back of the hitting cage as a buffer zone for golf ball flight, and a sponge pad was placed at the bottom of the hitting cage to prevent ball rebound upon impact, as shown in Figure 1.

### Test procedure

**Figure 2:**
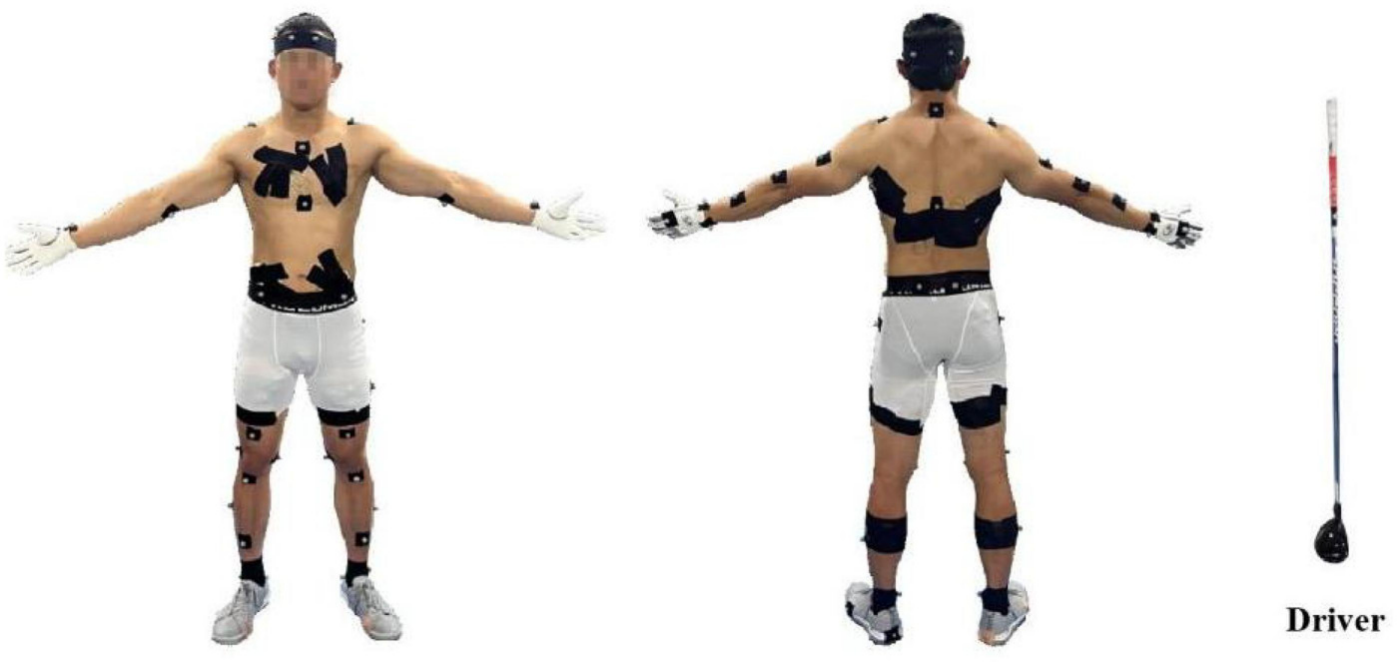
Marker placement protocol.

Each subject was instructed to warm up for 5 min on a treadmill at 6.5 km/h (25), followed by 5 min of swing practice using a driver. Prior to the experiment, testing equipment calibration was performed. Referencing Vicon's built-in Plug-In Gait 39-point full-body model marker placement scheme, and considering the technical characteristics of the golf full swing, additional tracking points were added to the subject's upper arm, forearm, both hands, as well as the clubhead and grip of the driver. A total of 54 reflective markers were affixed to collect kinematic data during the swing motion for both the subject's dominant and non-dominant sides, the marking positioning is shown in Figure 2 and Table 2. Prior to formal ball strikes, subjects stood in anatomical position on the 3D force platform for static data collection. Subsequently, they executed swings with a driver upon command. Radar equipment determined whether balls landed in-bounds. Five valid swing data sets per subject were recorded as valid results.

**Table 2:** Marker point name and paste location.

Marker name	Paste location	Marker name	Paste location
LFHD	Left forehead	RASI	Right anterior superior iliac spine
RFHD	Right forehead	LPSI	Left posterior superior iliac spine
LBHD	Left occipital region	RPSI	Right posterior superior iliac spine
RBHD	Right occipital region	LTROC	Left greater trochanter
C7	Spinous process of the 7th cervical vertebra	RTROC	Right greater trochanter
CLAV	xiphoid process	LFP1-2	Two left thigh tracking markers
STRN	Manubrium of the sternum	RFP1-2	Two right thigh tracking markers
T10	Spinous process of the 10th thoracic vertebra	LLTP	Left lateral femoral condyle
LSHO	Left acromion	RLTP	Right lateral femoral condyle
LUPA	Left humerus tracking marker	LMTP	Left medial femoral condyle
LELB	Left lateral epicondyle of the humerus	RMTP	Right medial femoral condyle
LEMB	Left medial epicondyle of the humerus	LTA1	Left knee marker
LUPB	Left forearm tracking marker	RTA1	Right knee marker
LWRA	Left radial styloid process	LTP2-3	Two left shank tracking markers
LWRB	Left distal ulna	RTP2-3	Two Right shank tracking markers
LFIN	Left 3rd metacarpal	LANK	Left lateral malleolus
RSHO	Right acromion	RANK	Right lateral malleolus
RUPA	Right humerus tracking marker	LMMA	Left lateral malleolus
RELB	Right lateral epicondyle of the humerus	RMMA	Right lateral malleolus
REMB	Right medial epicondyle of the humerus	LHEE	Left heel
RUPB	Right forearm tracking marker	RHEE	Right heel
RWRA	Right radial styloid process	LMT5	Left 5th metatarsophalangeal joint
RWRB	Right distal ulna	RMT5	Right 5th metatarsophalangeal joint
RFIN	Right 3rd metacarpal	LMT1	Left 1st metatarsophalangeal joint
LASI	Left anterior superior iliac spine	RMT1	Right 1st metatarsophalangeal joint

### Event division

**Figure 3:**
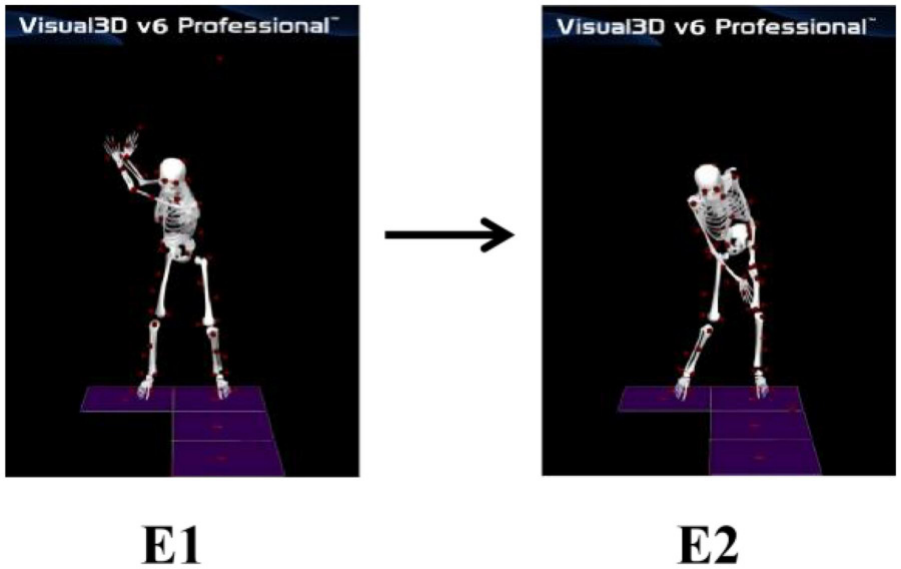
Division of swing phases.

The downswing phase was defined as the interval from the top of the backswing (E1) to impact (E2), as shown in Figure 3. Both events were identified manually based on the trajectory of the clubhead marker. E1 was defined as the frame at which the clubhead marker reached the end of the backswing and reversed direction toward the downswing. E2 was defined as the frame corresponding to club-ball contact, determined frame by frame from the clubhead marker trajectory relative to the ball position (9). To eliminate individual variations in downswing duration, all marker point trajectories were normalized to a 100% time series.

Top of backswing (E1): After the setup action is completed, the club begins to move to the maximum amplitude of the upper pole.

Impact (E2): The club continues to accelerate downward until the moment of impact.

Downswing Phase: From the top of backswing to the moment of impact, E1-E2.

### Data processing

Model preprocessing was performed using Vicon Nexus 2.9.1. Data were exported in C3D format and imported into Visual3D (C-Motion, USA) to establish a full-body skeletal model. Marker trajectories were low-pass filtered using a fourth-order zero-phase Butterworth filter with a cutoff frequency of 15 Hz (26, 27). Joint angles and angular velocities were then calculated in Visual3D, after which the processed data were organized and joint coordination calculations were performed using MATLAB R2024a (MathWorks, USA).

During kinematic data calculation, joint coordinate systems and joint angles were defined. The joint coordinate system comprised three fundamental planes of the participant's body: the sagittal plane, frontal plane, and horizontal plane. Joint movements within this coordinate system were defined as follows: Shoulder joint (X-sagittal plane: flexion/extension; Y-frontal plane: adduction/abduction; Z-Horizontal plane, internal/external rotation), elbow joint (X-Sagittal plane, flexion/extension; Z-Horizontal plane, internal/external rotation), wrist joint (X-Sagittal plane, flexion/extension; Y-Frontal plane, radial/ulnar deviation). Joint angle calculations are based on joint coordinate systems and sequences, with each joint angle corresponding to the relative motion of specific segments: the shoulder joint describes the movement of the upper arm relative to the trunk, the elbow joint describes the movement of the forearm relative to the upper arm, and the wrist joint describes the movement of the hand relative to the forearm.

### Joint coordination and variability calculation

Based on the technical characteristics of the golf swing, upper limb physiological structure, and biomechanical models (5, 6), this study compared the coordination patterns and variability of the shoulder-elbow, elbow-wrist, and shoulder-wrist joints in the sagittal plane on the left and right sides of the subjects' bodies, the shoulder-wrist joint coordination pattern and its variability in the frontal plane, and the shoulder-elbow joint coordination pattern and its variability in the horizontal plane. All biomechanical data from the downswing phase were first time-normalized to 101 data points (0%–100% of the motion cycle) for each valid trial, aiming to reduce the influence of inter-individual differences in downswing duration and enable point-by-point comparison of coordination waveforms across participants. Subsequently, for each participant, CRP was calculated for each individual trial. SPM original code in MATLAB procedure was used: five trial-specific CRP waveforms corresponding to a given coupling were aggregated to generate a representative subject-level CRP waveform for subsequent group-level SPM analysis. VCRP was computed by the MATLAB procedure as the across-trial standard deviation of the five CRP waveforms at each normalized time point, yielding a subject-specific VCRP waveform for each coupling that was further incorporated into group-level SPM analysis. Joint coordination and variability were assessed using CRP and VCRP (28), as shown in Table 3. In the equations, $\theta_{i}^{{\;}^{\prime }}$ is the normalized joint angle, *θ_i_* is the joint angle, *i* is the data point in the motion cycle, $\omega_{i}^{{\;}^{\prime }}$ is the normalized angular velocity, and *ω_i_* is the angular velocity. The normalized angle and angular velocity were calculated by Equations (1) and (2).

$$\begin{array}{c} \theta_{i}^{\;\prime }\;=\;\frac{2\cdot [\theta_{i}-\min(\theta_{i})]}{\max(\theta_{i})-\min(\theta_{i})}-1 \end{array}$$ (1)

$$\begin{array}{c} \omega_{i}^{\;\prime }\;=\;\frac{\omega_{i}}{\max[\max(\omega_{i}),\max(-\omega_{i})]} \end{array}$$ (2)

**Table 3:** Explanation of CRP values and slopes under different conditions.

CRP value and curve		Interpretation	
0		In phase (Joints in same angle and angular velocity)	
±π (3.1416)		Anti phase (Opposite angle and angular velocity)	
>0	Slope is 0	Proximal joint was leading	The fixed lead
>0	Positive slope		Expanding the lead
>0	Negative slope		Shrinking the lead
<0	Slope is 0	Distal joint was leading	The fixed lead
<0	Positive slope		Shrinking the lead
<0	Negative slope		Expanding the lead

Note: All values listed in the table are radian values converted from degrees.

The phase angle was then calculated using Equation (3), and the relative phase angle was calculated by Equation (4), subtract the distal phase angle from the proximal phase angle.

$$\begin{array}{c} \phi (i)\;=\;\tan^{-1}\left(\frac{\omega^{\;\prime }(i)}{\theta^{\;\prime }(i)}\right) \end{array}$$ (3)

$$\begin{array}{c} \mathrm{CRP}(i)=\Phi_{\;proximal}(i)-\Phi_{distal}(i) \end{array}$$ (4)

The CRP variability was calculated by Equation (5), indicating the standard deviation of all data points of the CRP value (VCRP).

$$\begin{array}{c} \mathrm{VCRP}=\frac{\sum_{i=1}^{N}SD_{i}}{N} \end{array}$$ (5)

The standard deviation was calculated using Equation (6).

$$SD_{i}=\sqrt{\frac{1}{n-1}\sum_{i=1}^{n}\left(x_{i}-\bar{x}\right)}$$ (6)

### Statistical analysis

Angular position and angular velocity time-series data for joint movements were first time-normalized prior to analysis, and the normality of scalar variables was assessed using the Shapiro–Wilk test. Since the data met the normality assumption, statistical analyses were performed using statistical parametric mapping (SPM). Because CRP and VCRP are continuous time-varying waveforms, SPM was used to compare the entire normalized downswing phase between the low handicap and high handicap groups rather than selected discrete variables (29). Independent-samples t-tests were conducted using spm1d (29, 30). All SPM analyses were performed using the open-source software package spm1d 0.4 within MATLAB (R2024a; The MathWorks, Inc., Natick, MA, USA). To control the family-wise error rate across the 12 separate SPM comparisons, Bonferroni correction was applied, and the corrected significance threshold was set at α = 0.004. For significant findings, Cohen's d was reported as an effect size index for the overall between-group difference.

## Results

### Shoulder-elbow joint coordination and its variability

**Figure 4:**
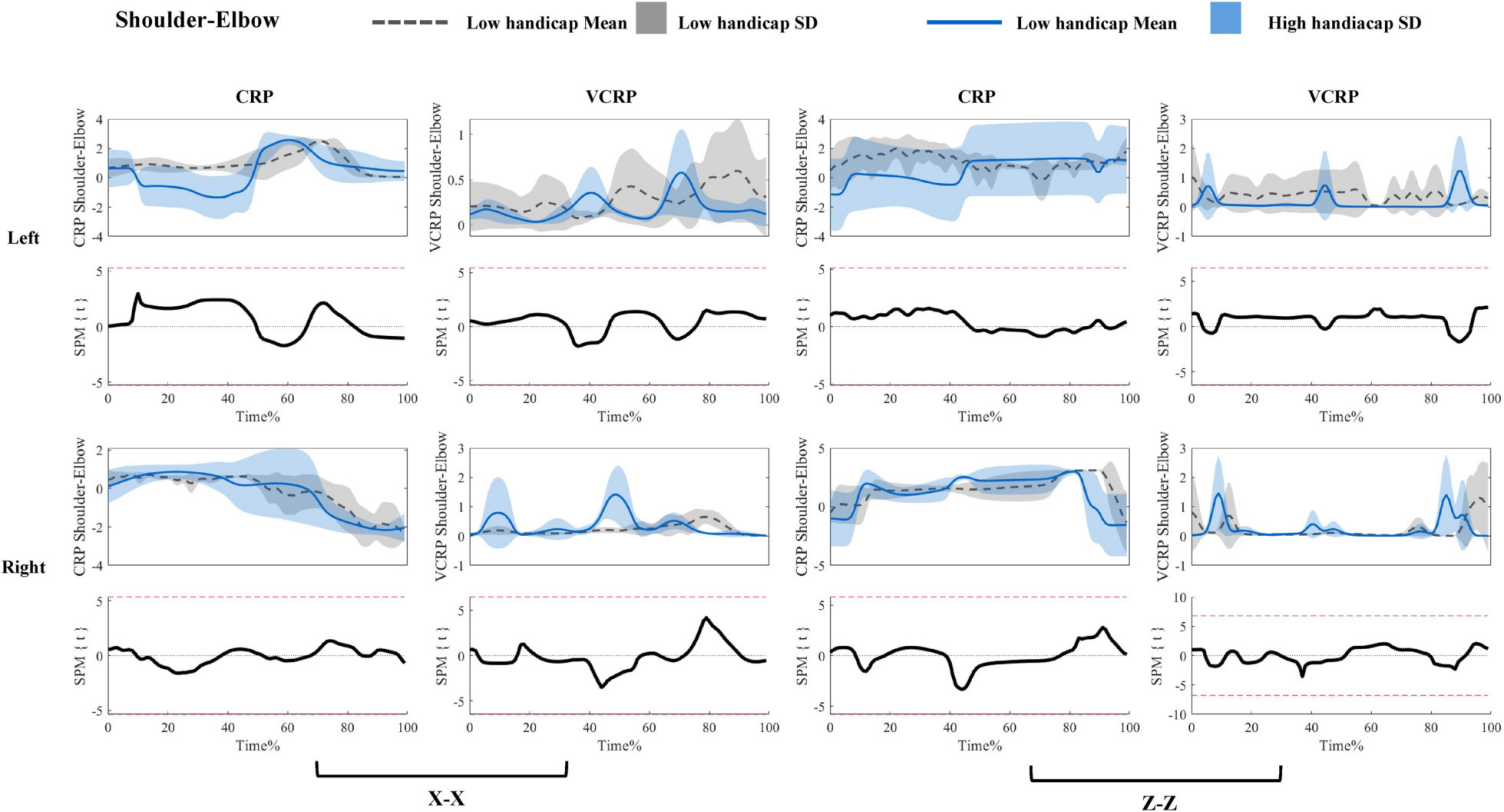
Joint coordination and variability of shoulder-elbow along the X, Z axis. The red dashed line indicates the critical threshold, the gray dashed area represents the over-threshold clustering region.

As shown in Figure 4, no significant differences were found for the following couplings during the downswing phase: left shoulder joint (horizontal)—left elbow joint (horizontal), right shoulder joint (sagittal)—right elbow joint (sagittal), and right shoulder joint (horizontal)—right elbow joint (horizontal). Independent samples *t*-tests revealed no clusters exceeding the threshold (*p* > 0.05).

### Elbow-wrist joint coordination and its variability

**Figure 5:**
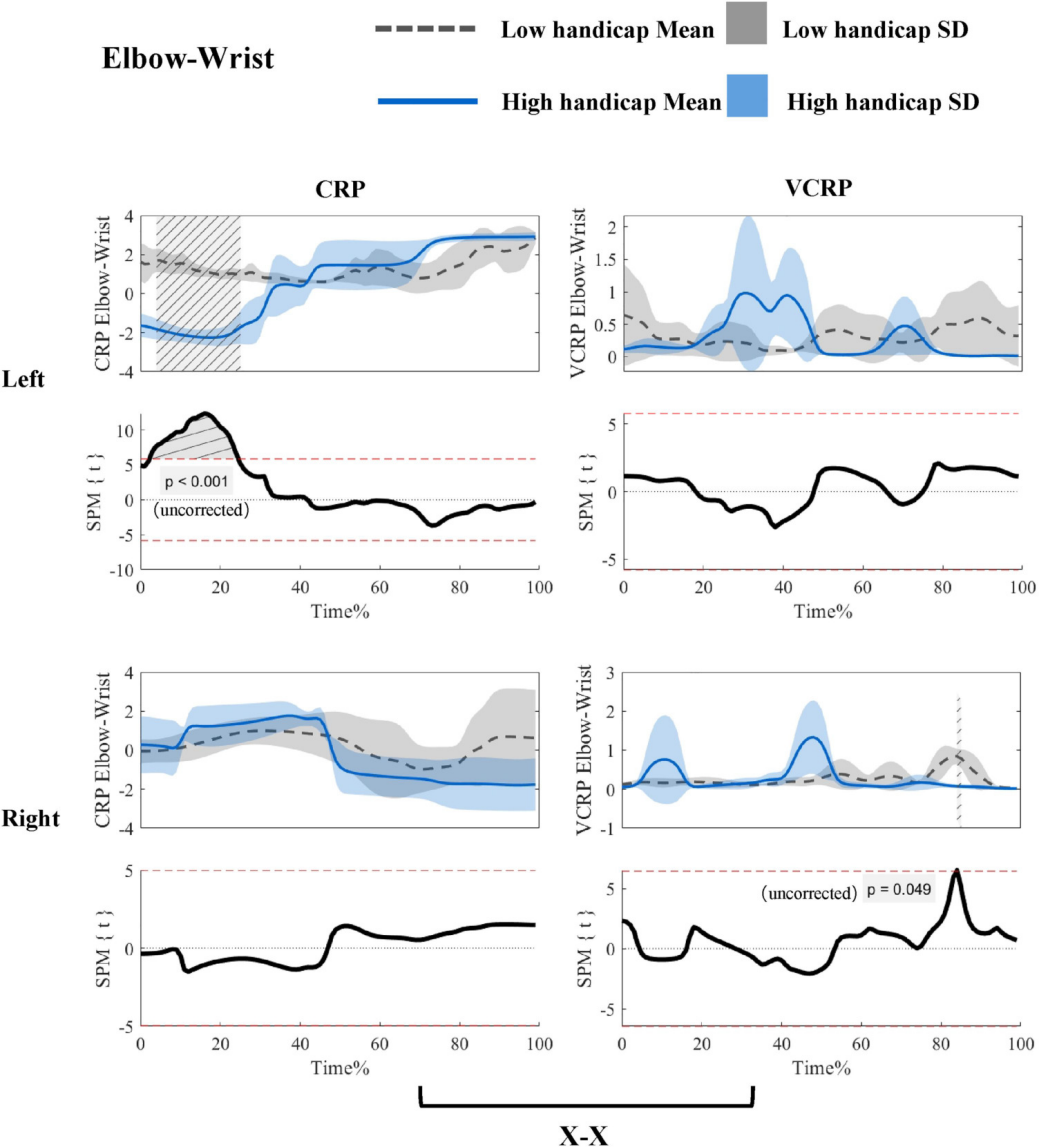
Joint coordination and variability of elbow-wrist along the X axis. The red dashed line indicates the critical threshold, the gray dashed area represents the over-threshold clustering region. *P* values shown in the figure are uncorrected. The significant cluster in the left CRP panel remained statistically significant after Bonferroni correction, whereas the significant cluster in the right VCRP panel did not remain significant after Bonferroni correction.

As shown in Figure 5, the CRP curve for the left elbow joint (sagittal)-left wrist joint (sagittal) coupling, a significant supra-threshold cluster was observed from 4% to 25% of the downswing phase. During this interval, the low handicap group showed positive CRP values, whereas the high handicap group showed negative CRP values, indicating different coordination tendencies between groups. The maximum t-statistic within this cluster was tmax = 12.36, exceeding the critical threshold (t* = 5.84), with an uncorrected *p* < 0.001 and a large effect size (Cohen's d = 2.043). This finding remained statistically significant after Bonferroni correction (α = 0.004). The VCRP curve of the right elbow joint (sagittal)-right wrist joint (sagittal) coupling, a significant supra-threshold cluster was observed from 84% to 85% of the downswing phase. The maximum t-statistic within this cluster was tmax = 6.53, exceeding the critical threshold (t* = 6.45), with an uncorrected *p* = 0.0494 and a trivial effect size (Cohen's d = −0.121). However, this effect did not remain significant after Bonferroni correction (α = 0.004). No other VCRP curves showed suprathreshold clusters exceeding the critical threshold (*p* > 0.05).

### Shoulder-wrist joint coordination and its variability

**Figure 6:**
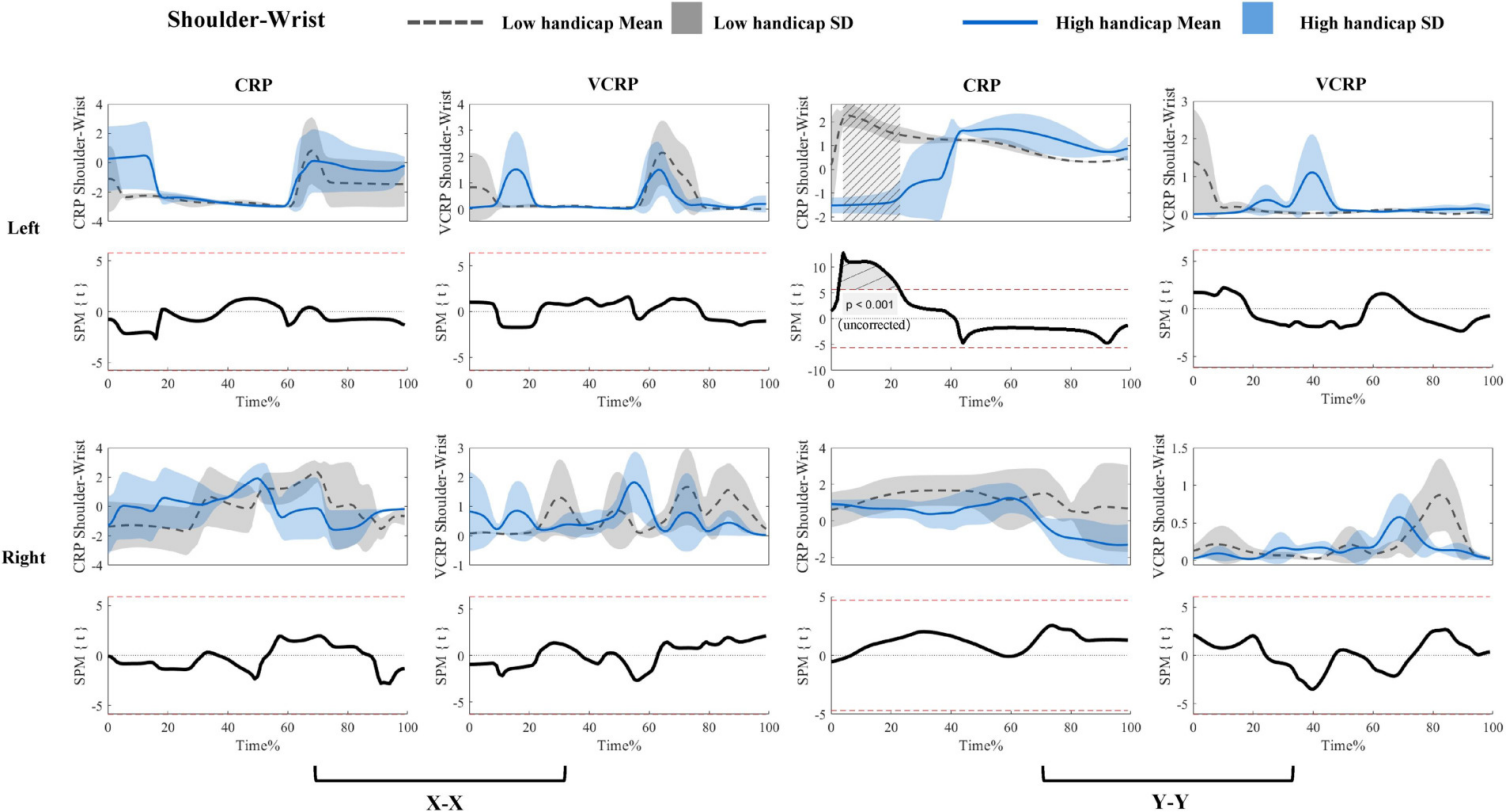
Joint coordination and variability of shoulder-wrist along the X, Y axis. The red dashed line indicates the critical threshold, the gray dashed area represents the over-threshold clustering region. *P* values shown in the figure are uncorrected. The significant cluster in the right CRP panel remained statistically significant after Bonferroni correction.

As shown in Figure 6, the CRP and VCRP curves during the downswing phase at the shoulder joint (sagittal)—wrist joint (sagittal) did not reveal clusters exceeding the threshold (*p* > 0.05). In the CRP curve for the left shoulder joint (frontal)-left wrist joint (frontal) coupling, the low handicap group exhibited higher CRP values than the high handicap group from 4% to 23% of the downswing phase. The maximum t-statistic within this cluster was tmax = 12.72, exceeding the critical threshold (t* = 5.63), with an uncorrected *p* < 0.001 and a large effect size (Cohen's d = 1.802). This result remained statistically significant after Bonferroni correction (α = 0.004). No other CRP or VCRP curves showed suprathreshold clusters exceeding the critical threshold (*p* > 0.05).

## Discussion

This exploratory study aimed to examine differences in upper limb joint coordination and coordination variability between golfers with low and high handicaps during the downswing phase of the golf swing. Results indicated differences in joint coordination between the low and high handicap groups in the coupling of the left elbow joint (sagittal)—left wrist joint (sagittal), as well as the coupling of the left shoulder joint (frontal)—left wrist joint (frontal). In addition, a between-group difference in coordination variability was observed in the right elbow joint (sagittal)-right wrist joint (sagittal) coupling at the uncorrected level. Overall, the findings suggest that the low handicap and high handicap groups differed in specific upper-limb coordination patterns during the downswing, whereas the coordination-variability finding should be interpreted more cautiously. Given the limited application of CRP/VCRP analysis in golf, these findings should be interpreted as exploratory and hypothesis-generating.

### Inter-joint coordination

In the present study, significant differences in upper-limb joint coordination patterns were observed between the low handicap and high handicap groups during the early downswing phase (4%–25%), primarily in the sequence and direction of joint movements. Regarding the coupling between the left elbow joint (sagittal)—left wrist joint (sagittal), the low handicap group exhibited a proximal-leading pattern dominated by the elbow joint, while the high handicap group demonstrated a distal-leading pattern dominated by the wrist joint. The proximal-dominant leading pattern with the elbow joint as the primary driver indicates that, in the early stage of the downswing, the elbow joint of the low handicap group leads the wrist joint in terms of motion timing, suggesting that the elbow joint plays an earlier organizing or driving role in this coupling. Existing biomechanical research on golf suggests that an efficient downswing generally follows a proximal-to-distal temporal organization (8). From a mechanical perspective, a relatively leading role of the elbow joint-rather than a premature dominance of the wrist joint-in the upper-limb sequence may be more conducive to effective speed generation (8). However, it should be noted that empirical studies directly examining the elbow-wrist joint timing relationship and its impact on clubhead speed remain relatively limited. Moreover, as this study did not involve relevant kinetic data, the interpretation of the results should be approached with caution. Additionally, the findings of this study differed somewhat from those of Liu (5), who identified a new pattern in the upper-limb biomechanics of sports such as baseball and javelin throwing that deviates from the traditional “shoulder-elbow-wrist” joint sequence, namely “shoulder-elbow-shoulder-wrist”. The discrepancies between the results of this study and those of Liu (5) may be attributed to: first, technical differences among various sports; and second, differences in research objectives, with Liu's results reflecting the diversity of biomechanical strategies in executing technical movements.

The results also showed that the high handicap group exhibited a progressively increasing tendency toward counterphase coupling over time during the movement. In the present study, counterphase coupling refers to a coordination pattern in which adjacent joints exhibit opposite phase tendencies, rather than moving in a more synchronized temporal relationship (31). More specifically, counterphase coupling describes a pattern in which adjacent joints move in opposite directions at the same time, such as elbow extension occurring simultaneously with wrist flexion, in contrast to in-phase coupling, where the joints move in the same direction. In this context, the stronger tendency toward counterphase coupling in the high handicap group may reflect the adoption of a different inter-joint coordination strategy during the early downswing. However, this finding should still be interpreted with caution. Although previous studies have suggested that counterphase coupling patterns may increase joint stress and torsional loading, thereby contributing to injury risk (32–34), the present study did not collect kinetic data, joint loading measures, or injury-related variables. Therefore, injury risk cannot be directly inferred from the current findings alone. Future research should integrate inverse dynamics modeling and longitudinal injury surveillance to clarify the relationship between coordination variability and joint loading.

Additionally, during the early downswing phase (4%–23%), differences emerged between the low handicap and high handicap groups in the coupling between the left shoulder joint (frontal)—left wrist joint (frontal). The low handicap group exhibited a shoulder-dominant proximal joint shortening-leading coupling pattern, where the shoulder joint initiated and dominated as the proximal segment, with dominance gradually shifting distally (toward the wrist joint). In contrast, the high handicap group exhibited a coordination pattern dominated by distal wrist joint fixation. This implies that during the initial phase of the movement, the distal segment prematurely and persistently assumed a dominant role in the temporal sequence. Studies by Egret et al. (35) and Brétigny et al. (36) suggest that this delay in movement from proximal to distal segments facilitates the gradual transfer of kinetic energy, creating conditions for the subsequent “release” phase. Concurrently, studies by Fedorcik et al. (37) and Carson et al. (38) suggest this movement pattern is closely linked to the “delayed ulnar deviation” phenomenon in the lead wrist (left wrist for right-handed players) among golfers with low handicaps, representing a key factor in enhancing athletic performance. Therefore, integrating the findings of this study, the coordination pattern dominated by fixation of the left wrist (frontal plane) in the high handicap group may reflect an absence of this delay mechanism. Premature activation of the wrist joint, which remains the dominant joint throughout the motion, was associated with less effective potential energy storage and may reflect reduced accumulation of terminal velocity (39). Notably, while Carson et al. (38) confirmed the common kinematic characteristics of the left wrist in golfers with low handicaps, they found no significant differences in clubhead speed across different grip techniques. This may indicate that for highly skilled players who have mastered the technical movements, proximal joint dominance has become an inherent, fundamental coordination pattern. In contrast, the proximal advantage pattern exhibited by golfers with high handicaps may represent a secondary coordination strategy—a compensation method that reflects excessive reliance on distal force or control when coordination ability is insufficient. However, this explanation should still be treated with caution, as this study did not collect kinetic data and other explanations cannot be ruled out.

Overall, compared to the low handicap group, the high handicap group exhibited differences in upper-body coordination patterns during the early phase of the downswing in terms of both sequence and direction. In terms of sequence, this manifested as earlier involvement of the wrist joint, which may be less consistent with the proximal-to-distal pattern commonly observed in skill-based striking tasks. In terms of direction, it manifested as a more pronounced tendency toward counter-phase movement between adjacent joints. However, these findings should not be interpreted as the direct cause of the high handicap group's poorer performance. Rather, they more likely represent coordination characteristics associated with skill level, while alternative explanations-such as strength, movement speed, technical background, and task constraints-cannot be ruled out. Therefore, the results of this study can, at most, provide preliminary guidance for future coaching practices and intervention studies, but cannot directly serve as a basis for training prescriptions.

### Coordination variability

This study found that during the late downswing phase (83%–85%), the low handicap group exhibited a transient peak in the VCRP curve between the right elbow (sagittal)—right wrist (sagittal) joint. However, because this significant interval was short, and the result did not remain statistically significant after Bonferroni correction, it was not yet sufficient to regard it as a robust between-group difference. A more appropriate interpretation is to treat it as a preliminary signal observed under uncorrected conditions. This finding suggests that variability in elbow–wrist coordination during the late downswing may have some research value, but its specific significance still needs to be further verified in future studies with larger samples, sensitivity analyses, and kinetic variables.

## Limitations

Several limitations should be acknowledged. First, the sample size was relatively small, particularly given the number of SPM comparisons performed. Second, only five valid trials per participant were included, which may limit the stability of the VCRP estimates. Third, the study used a cross-sectional design, so the observed differences cannot be interpreted causally. Fourth, only young male golfers were included, which limits the generalizability of the findings to female golfers or other age groups. Fifth, the present analysis focused exclusively on upper-limb coordination during the downswing and did not incorporate other phases of the swing, lower-limb mechanics, trunk motion, ground reaction forces, or kinetic variables. Finally, the VCRP finding was confined to a very narrow time window and did not remain statistically significant after Bonferroni correction; therefore, its functional interpretation should be treated with caution.

## Conclusion

This study employed statistical parametric mapping to investigate differences in upper limb joint coordination and coordination variability between golfers with low and high handicaps during the downswing phase. The main conclusions were as follows:

(1) Regarding joint coordination, the high handicap group exhibited earlier wrist engagement in the early downswing and a more pronounced tendency toward inverse motion between adjacent joints.
(2) Regarding coordination variability, the high handicap group exhibited lower variability in elbow-wrist joint coupling during a brief interval at the end of the downswing. However, since this result was no longer significant after Bonferroni correction, it should be interpreted with caution as a preliminary finding.

## Future direction

Given the exploratory nature of this study, the following hypotheses are generated for future investigation:

(1) We hypothesize that training interventions focused on strengthening proximal joint initiation may improve coordination patterns in golfers with high handicaps. However, this hypothesis requires testing in future intervention studies.
(2) We further hypothesize that shifting training focus from rigid postures to dynamic coordination regulation may enhance coordination adaptability in golfers with high handicaps, a prediction that awaits empirical validation.

Future longitudinal or intervention studies with larger, more diverse samples are needed to determine whether the patterns observed in this exploratory study replicate across populations and whether modifying these patterns causally affects performance or injury risk.

## Data Availability

The raw data supporting the conclusions of this article will be made available by the authors, without undue reservation.
